# Secoisolariciresinol Diglucoside Abrogates Oxidative Stress-Induced Damage in Cardiac Iron Overload Condition

**DOI:** 10.1371/journal.pone.0122852

**Published:** 2015-03-30

**Authors:** Stephanie Puukila, Sean Bryan, Anna Laakso, Jessica Abdel-Malak, Carli Gurney, Adrian Agostino, Adriane Belló-Klein, Kailash Prasad, Neelam Khaper

**Affiliations:** 1 Department of Biology, Lakehead University, Thunder Bay, Ontario, Canada; 2 Northern Ontario School of Medicine, Lakehead University, Thunder Bay, Ontario, Canada; 3 Department of Physiology, Federal University of Rio Grande do Sul, Porto Alegre, Rio Grande do Sul, Brazil; 4 Department of Physiology, College of Medicine, University of Saskatchewan, Saskatoon, Saskatchewan, Canada; University of Central Florida, UNITED STATES

## Abstract

Cardiac iron overload is directly associated with cardiac dysfunction and can ultimately lead to heart failure. This study examined the effect of secoisolariciresinol diglucoside (SDG), a component of flaxseed, on iron overload induced cardiac damage by evaluating oxidative stress, inflammation and apoptosis in H9c2 cardiomyocytes. Cells were incubated with 50 μ5M iron for 24 hours and/or a 24 hour pre-treatment of 500 μ M SDG. Cardiac iron overload resulted in increased oxidative stress and gene expression of the inflammatory mediators tumor necrosis factor-α, interleukin-10 and interferon γ, as well as matrix metalloproteinases-2 and -9. Increased apoptosis was evident by increased active caspase 3/7 activity and increased protein expression of Forkhead box O3a, caspase 3 and Bax. Cardiac iron overload also resulted in increased protein expression of p70S6 Kinase 1 and decreased expression of AMP-activated protein kinase. Pre-treatment with SDG abrogated the iron-induced increases in oxidative stress, inflammation and apoptosis, as well as the increased p70S6 Kinase 1 and decreased AMP-activated protein kinase expression. The decrease in superoxide dismutase activity by iron treatment was prevented by pre-treatment with SDG in the presence of iron. Based on these findings we conclude that SDG was cytoprotective in an *in vitro* model of iron overload induced redox-inflammatory damage, suggesting a novel potential role for SDG in cardiac iron overload.

## Introduction

Iron is essential to biochemical, metabolic, and biological processes in all organisms, where it is the critical component of haemoglobin and is needed for energy production and detoxification [1]. However, an overabundance of iron can result in complications such as cardiomyopathy, cirrhosis, and diabetes [2–6]. Primary iron overload, or hemochromatosis, is a common autosomal recessive disorder where mutation of the hemochromatosis-associated gene causes impaired feedback inhibition of iron uptake, resulting in maximal absorption [5,6]. Secondary iron overload typically derives from dietary, transfusion excesses, iron-loading anemia, and chronic liver diseases [1, 4–6].

Chronic iron overload can lead to a variety of cardiac arrhythmias ultimately resulting in heart failure [2, 5–8]. Although no single mechanism is likely to account for the pathology of iron-overload induced heart failure, recent studies have suggested that altered calcium homeostasis and increased oxidative stress each play a role [5–8]. The mechanism of cardiac iron uptake is not well defined but recent studies have established that iron is transported by voltage-dependent L-type Ca^2+^ channels in cardiomyocytes [2, 6, 8].

A significant relationship exists between oxidative stress, inflammation and apoptosis in the pathophysiology of cardiovascular diseases, whereby increased ROS have been shown to promote pro-inflammatory mediator expression [9–11]. Forkhead box O (FOXO)3a is a transcription factor that promotes cardiomyocyte survival upon induction of oxidative stress [12]. TNF-α can trigger the expression and activation of matrix metalloproteinases (MMP)s via superoxide production [13]. Oxidative stress has been implicated in cardiac remodeling by causing an increase in activation of p70S6 Kinase 1 (p70S6K1) [14]. Studies have shown AMP-activated protein kinase (AMPK) can protect the heart from ischemic injury and adverse cardiac remodeling [15], in part via inhibition of p70S6K1 [16].

Once cardiac dysfunction is detected, the prognosis is poor without intervention but can be improved if appropriate therapy is given to address the iron overload [5, 6]. Studies have also found that free iron can rapidly transfer from extracellular medium into the mitochondria and can therefore be unreachable by chelators [17]. Given these limitations, novel methods for abrogating iron overload-induced damage that target key pathological processes including oxidative stress and inflammation are very desirable.

Secoisolariciresinol diglucoside (SDG) is a phytochemical antioxidant present in flaxseed. It has been shown to decrease the production of inflammatory mediators and reduce oxidative stress [18–20]. SDG treatment was also found to reduce the development of hypercholesterolemic atherosclerosis in rabbits fed a high cholesterol diet [21], and reduce the development of diabetes in rats [22, 23]. However, there have been no studies to date investigating the role of SDG in oxidative damage in a cardiac iron overload model.

The present study was designed to investigate the cytoprotective effects of SDG on iron overload induced redox-inflammatory changes using the H9c2 cell line. Cellular damage was evaluated in terms of oxidative stress and inflammation and was correlated with apoptosis.

## Materials and Methods

### Cell culture methods

Cardiac (H9c2) cells were obtained from American Type Culture Collection (Manassas, VA, USA) and cultured in 25 cm^3^, 75 cm^3^ and 150 cm^3^ cell culture flasks as per the recommended protocol: Dulbecco’s Modified Eagle’s Medium (DMEM) (Sigma-Aldrich, St. Louis, MO, USA) with 10% fetal calf serum (Hyclone, Pittsburgh, PA, USA), 1% penicillin-streptomycin (Invitrogen, Carlsbad, CA, USA) at 37°C, 5% CO_2_ and 100% humidity. Cells were seeded in an appropriate amount of medium and allowed to adhere 24 hours prior to treatment exposure.

### Iron and SDG preparation

50 μM iron solution was prepared by dissolving ammonium iron (III) citrate (Sigma-Aldrich, St. Louis, MO, USA) into serum- and antibiotic-free DMEM. A pre-treatment of 500 μM SDG (kindly provided by Dr. K Prasad) was prepared by dissolving SDG into serum- and antibiotic-free DMEM. For each treatment cells were incubated for 24 hours at 37°C, 5% CO_2_ and 100% humidity.

### Cell surface area

Images were obtained with the Nikon Eclipse TS100 microscope at 100x magnification and captured with the Nikon DS-Fi1 (Version 4.20) camera (Nikon Instruments Inc. Melville, NY, USA). Cell size was measured using ImageJ from 10 images per flask per treatment and expressed as mm^2^.

### H_**2**_DCFDA (Oxidative Stress) assay

Oxidative stress in the form of intracellular ROS was determined via the H_2_DCFDA assay (Life Technologies, Carlsbad, CA, USA). After a 24-hour iron treatment ~7–8 x 10^6^ cells were washed with phosphate buffer saline (PBS) and incubated for 30 minutes with H_2_DCFDA according to the manufacturer’s instructions. The H_2_DCFDA was then aspirated and the cells were washed with PBS. The cells were then carefully removed from the culture dish by trypsinization and the relative fluorescence of untreated and iron-treated cells was measured via flow cytometry (Becton Dickinson FACSCalibur flow cytometer) as per the manufacturer’s instructions.

### Superoxide dismutase assay

Superoxide Dismutase (SOD) was measured using the Superoxide Dismutase Assay kit (Trevigen, Helgerman Ct. Gaithersburg, MD, USA). ~5 x 10^6^ cells were washed with PBS and carefully removed from the culture dish by trypsinization and centrifuged at 500 x g for 5 minutes. Pelleted cell samples were lysed and their total protein quantitated via *DC* Protein Assay (Bio-Rad Laboratories, Inc., Hercules, CA, USA). Samples were assayed as per the kit’s instructions and absorbance read at 550 nm at five minute interval using a PharmaSpec UV-1700 Visible Spectrophotometer (Shimadzu, Columbia, MD, USA). Data are expressed as SOD units per volume with reference to an SOD inhibition curve.

### Total RNA isolation and quantification

Total RNA was extracted from ~1 x 10^6^ cells using the Aurum Total RNA Mini Kit (Bio-Rad Laboratories, Inc., Hercules, CA, USA) as per the manufacturer’s instructions. RNA integrity and concentration were determined using the Experion semi-automated electrophoresis system (Bio-Rad Laboratories, Inc., Hercules, CA, USA).

### cDNA amplification and quantitative real-time PCR

A total of 1 μg total RNA was reverse-transcribed to cDNA using the First Strand cDNA Synthesis Kit (MBI Fermentas, Flamborough, ON, CAN) as per the manufacturer’s instructions. Quantitative real-time PCR reactions were performed using primers (SuperArray, Frederick, MD, USA) on an iQ5 iCycler (Bio-Rad Laboratories, Inc., Hercules, CA, USA) as per the manufacturer’s instructions: 1 cycle at 95°C / 10 min, followed by 45 cycles of 95°C / 15 seconds; 60°C / 60 seconds. All samples were normalized to β2-microglobulin expression. Relative fold increase was calculated as per the convention: change in sample cycle threshold with regard to reference gene expression. A single peak (or zero if no product was amplified) was present in the first-derivative dissociation curves for every PCR reaction on all arrays, indicating that only a single PCR product, the gene of interest, was amplified.

### CaspaTag (Apoptosis Detection) assay

Active caspase-3 and -7 were detected by the CaspaTag Caspase 3/7 assay (Chemicon International, Temecula, CA, USA) according to the manufacturer’s protocol. ~1 x 10^6^ cells were stained with Fluorochrome Inhibitors of Caspases (FLICA) and incubated for 1 hour at 37°C. Cells were mixed every 20 minutes during staining. FLICA was then aspirated and the cells were washed with 1X wash buffer. Active caspase-3/7 positive cells were assessed by flow cytometry based on their green fluorescence following incubation with carboxyfluorescein-labeled fluromethyl ketone peptide inhibitor of caspase-3. The activation of caspase-3/7 was expressed as the mean fluorescence of iron-treated relative to untreated cells.

### Western blot

Cells were homogenized in 200 μL of Nonidet P40 (NP-40) (Roche Diagnostics, Mannheim, Germany) buffer containing 150 mM NaCl, 1% NP-40, 50 mM Tris (pH 8.0) and protease inhibitors phenylmethylsulfonyl fluoride, leupeptin, aprotinin and pepstatin. Cell debris was removed by centrifugation at 12,000 x *g* for 30 minutes at 4°C, and the protein content determined by Bradford assay (Bio-Rad Laboratories, Inc., Hercules, CA, USA). 50 μg of protein from each sample was boiled and subjected to electrophoresis in denaturing 10% SDS-PAGE. Proteins were transferred to polyvinylidene fluoride (PVDF) membranes using a Bio-Rad Trans-blot apparatus. The membranes were blocked with 5% bovine serum albumin (BSA) for 1.5 hours at room temperature. After blocking, the membrane was incubated with the appropriate antibody overnight at 4°C. Following extensive washing in 1X Tris buffered saline (TBS; pH 7.4), the membrane was incubated with the appropriate horseradish peroxidase (HRP)-conjugated secondary antibody for 2 hours at room temperature. Finally, the membrane was washed extensively in 1X TBS, and protein expression was visualized using the enhanced chemiluminescence reagent. Following chemiluminescent imaging, membranes were stripped for re-blotting. β-actin (1:5000), Bax (1:100) and Bcl2 (1:100) antibodies were purchased from Santa Cruz Biotechnology, Santa Cruz, CA, USA. AMPK (1:1000), FOXO3a (1:1000) and p70S6K1 (1:1000) antibodies were purchased from Cell Signaling Technology, Danvers, MA, USA. The secondary antibodies were anti-rabbit IgG (1:5000; Cell Signaling Technologies, Danvers, MA, USA), used for all primary antibodies except for Bcl2, which used anti-mouse IgG (1:2000). The dilution was made in 5% BSA solution.

### Statistical analysis

Data were presented as mean ± SEM and all data represents n ≥ 3 independent experiments. Statistical analyses were performed using GraphPad Prism software. One-way ANOVA with post hoc Tukey’s test were utilized when possible with *p < 0*.*05* considered significant. Asterisks are used herein to denote significance according to the following scheme: * = *p < 0*.*05*; ** = *p < 0*.*01*; ***/### = *p < 0*.*001*.

## Results

### Effect of iron and SDG on cell surface area

**Fig 1:**
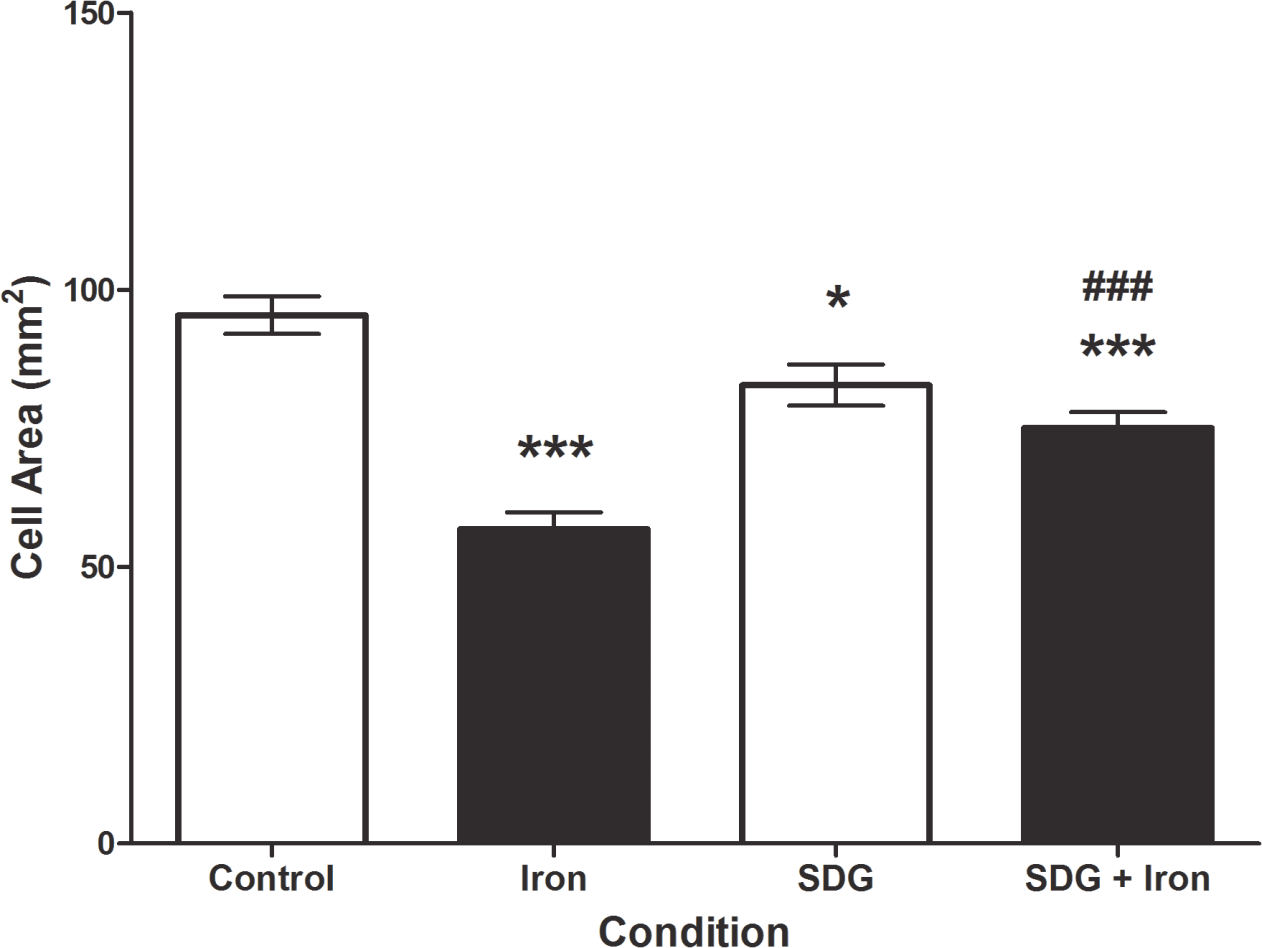
Pretreatment with SDG prevents iron induced decrease in H9c2 cell size. Cell surface area of control, 50 μM iron-treated, 500 μM secoisolariciresinol diglucoside (SDG), and SDG pre-treatment + iron-treated H9c2 cells. Cell surface area was assessed with ImageJ software. Data is expressed as mean cell area (mm^2^) (*** = p < 0.001 vs control; * = p < 0.05 vs control; ### = p < 0.001 vs iron, *n* = 10 images).

Cell surface area was calculated with ImageJ software. A 24-hour treatment with 50 μM iron caused a significant decrease in cell size (p < 0.001) when compared to control cells (Fig 1). 24-hour pre-treatment with 500 μM SDG caused cell size to decrease when compared to control but were still significantly larger than iron treated cells (p < 0.001) (Fig 1). These data indicate that pre-treatment with SDG can prevent cell size decrease caused by iron overload.

### Effect of SDG on iron-induced oxidative stress

**Fig 2:**
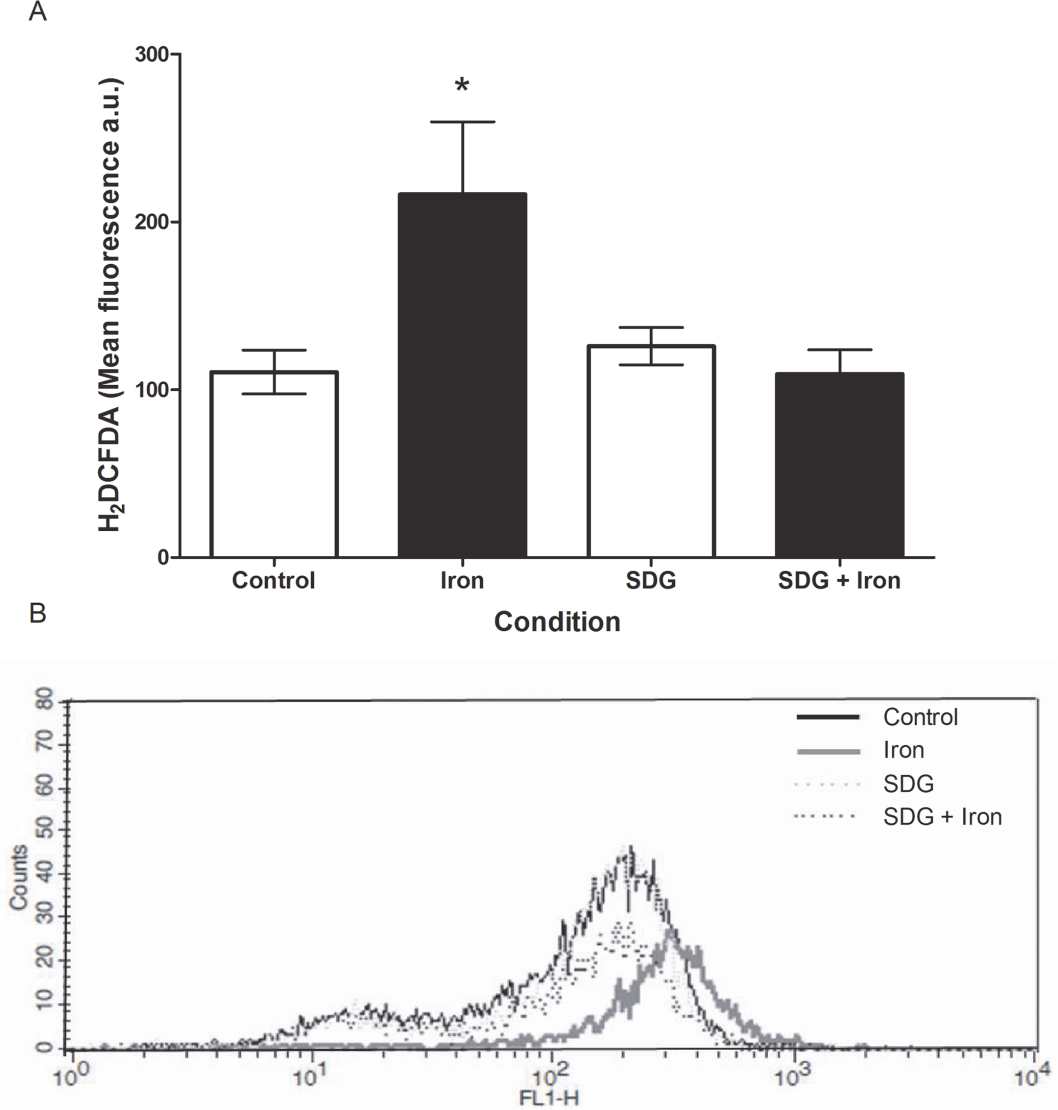
SDG decreases iron-induced oxidative stress in H9c2 cells. Reactive oxygen species (ROS) in control, 50 μM iron-treated, 500 μM secoisolariciresinol diglucoside (SDG), and SDG pre-treatment + iron-treated H9c2 cells. ROS levels were assessed using the CM-H_2_DCFDA assay and measured via flow cytometry (A). The representative histogram of cell count versus FL1 fluorescence (B). Data is expressed as mean fluorescence arbitrary units (a.u.) (* = p < 0.05 versus control, *n* = 3).

Intracellular ROS levels were assessed using the CM-H_2_DCFDA assay and measured via flow cytometry. A 24-hour treatment with 50 μM iron caused a significant increase (p < 0.05) in ROS generation (Fig 2*A*) as indicated by increased mean FL1 fluorescence (representative histogram; Fig 2*B*) versus control. These data indicate a pronounced production of ROS resulting from iron treatment of the H9c2 cells. 24-hour pre-treatment with 500 μM SDG prevented the significant increase in iron-induced ROS generation, reducing it to control levels (Fig 2*A* and 2*B*). This finding indicates that the observed iron-induced increase in ROS was attenuated by pre-treatment with SDG.

### Effect of SDG on SOD concentration

**Fig 3:**
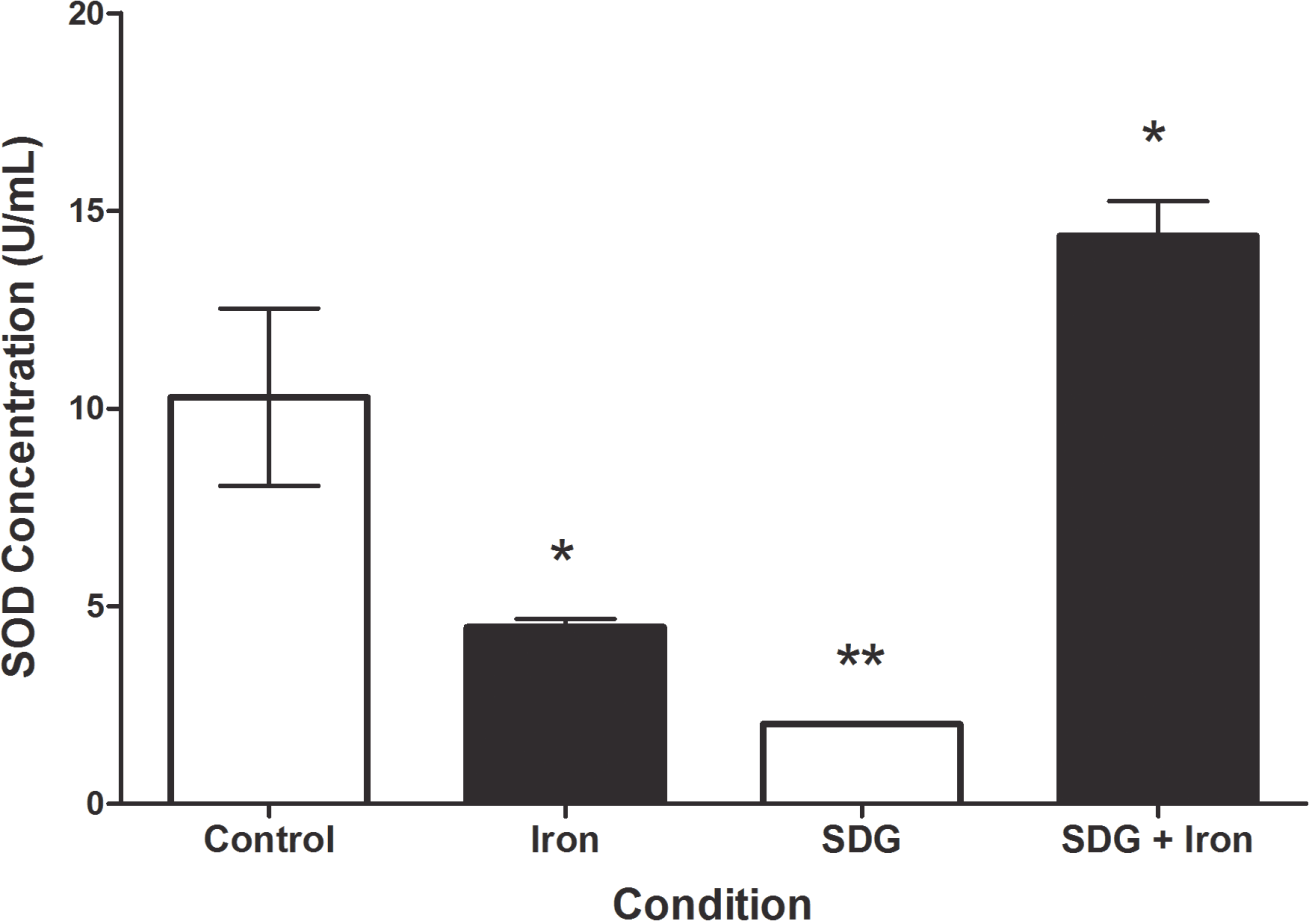
SDG increases SOD concentration in iron treated H9c2 cells. SOD concentration in control, 50 μM iron-treated, 500 μM secoisolariciresinol diglucoside (SDG), and SDG pre-treatment + iron-treated H9c2 cells. SOD concentration was assessed via a colourimetric assay. Data is expressed as SOD concentration (U/mL). (** = p < 0.01 versus control; * = p < 0.05 versus control, *n* = 3).

To investigate potential cellular antioxidant responses the concentration of SOD in iron and SDG treated cells was measured via the Superoxide Dismutase assay kit. Iron and SDG treatment caused a decrease in SOD concentration (p < 0.05 and p < 0.01, respectively) when compared to the control. Combined treatment with SDG and iron lead to an increase in SOD concentration when compared to the control (p < 0.05) (Fig 3). This finding suggests that iron-induced decrease in SOD activity can be abrogated by treatment with SDG.

### Effect of SDG on inflammatory cytokine expression

**Fig 4:**
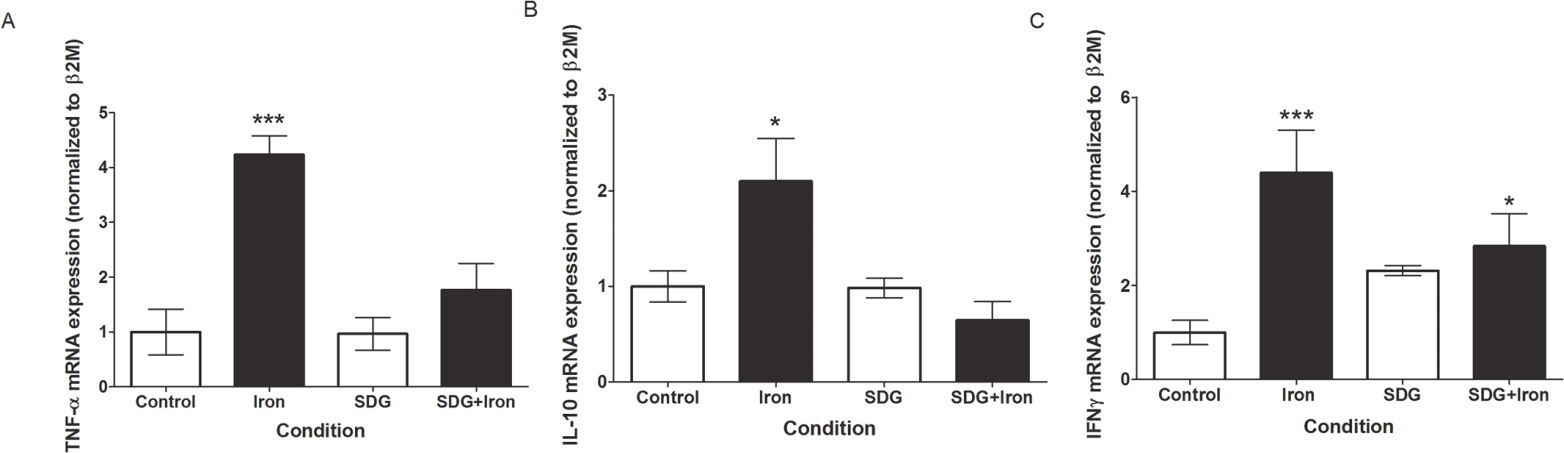
SDG decreases iron-induced inflammatory cytokine gene expression in H9c2 cells. Gene expression of inflammatory cytokines in control, 50 μM iron-treated, 500 μM secoisolariciresinol diglucoside (SDG), and SDG pre-treatment + iron-treated H9c2 cells. mRNA levels of tumor necrosis factor (TNF)-α (A), interleukin (IL)-10 (B), and interferon (IFN)γ (C) were determined via quantitative real-time PCR and normalized to β2-microglobulin, with normal expression standardized to the control. (*** = p < 0.001 versus control; * = p < 0.05 versus control, *n* = 3).

Given the established cross-promotional relationship of oxidative stress and inflammation, the effect of iron on the expression of TNF-α, IL-10, and IFNγ was investigated via qPCR. Iron treatment caused significant increases in TNF-α (~3.2 fold, p < 0.001) (Fig 4*A*) and IFNγ (~3.4 fold, p < 0.001) (Fig 4*C*) and a modest increase in IL-10 (~1.1 fold, p < 0.05) (Fig 4*B*), versus control. These data indicate greatly enhanced expression of key inflammatory mediators in response to iron treatment. Pre-treatment with SDG mitigated the significant increase in iron-induced TNF- α and IL-10 expression, maintaining it at control levels (Fig 4*A* and 4*B*). IFNγ expression increased in SDG + iron treated cell when compared to control cells (~2.8 fold, p < 0.05).

### Effect of SDG on MMP2 and 9 expression

**Fig 5:**
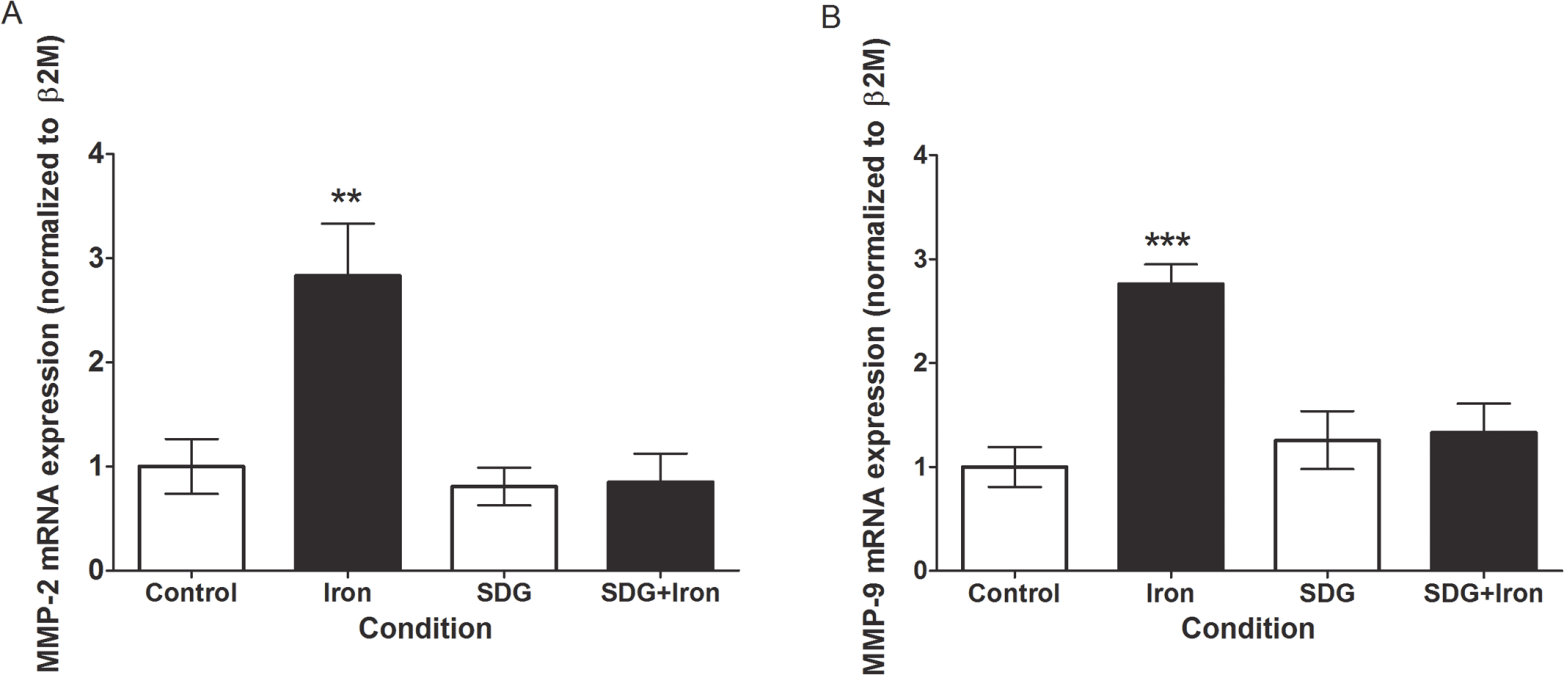
SDG decreases iron-induced MMP gene expression in H9c2 cells. Gene expression of matrix metalloproteinases in control, 50 μM iron-treated, 500 μM secoisolariciresinol diglucoside (SDG), and SDG pre-treatment + iron-treated H9c2 cells. mRNA levels of matrix metalloproteinase (MMP)-2 (A) and MMP-9 (B) were determined via quantitative real-time PCR and normalized to β2-microglobulin, with normal expression standardized to the control. (*** = p < 0.001 versus control; ** = p < 0.01 versus control, *n* = 3).

Since oxidative stress and inflammation are demonstrably linked to matrix degradation and cardiac remodelling, the expression of MMP-2 and 9 was investigated via qPCR. Iron treatment caused statistically significant increases in MMP-2 (~1.8 fold, p < 0.01) (Fig 5*A*) and MMP-9 (~1.7 fold, p < 0.001) (Fig 5*B*) versus control. Pre-treatment with SDG nullified the significant increase in iron-induced MMP-2 and 9 expression, maintaining it at control levels (Fig 5*A* and 5*B*).

### Effect of SDG on iron-induced cardiomyocyte apoptosis

**Fig 6:**
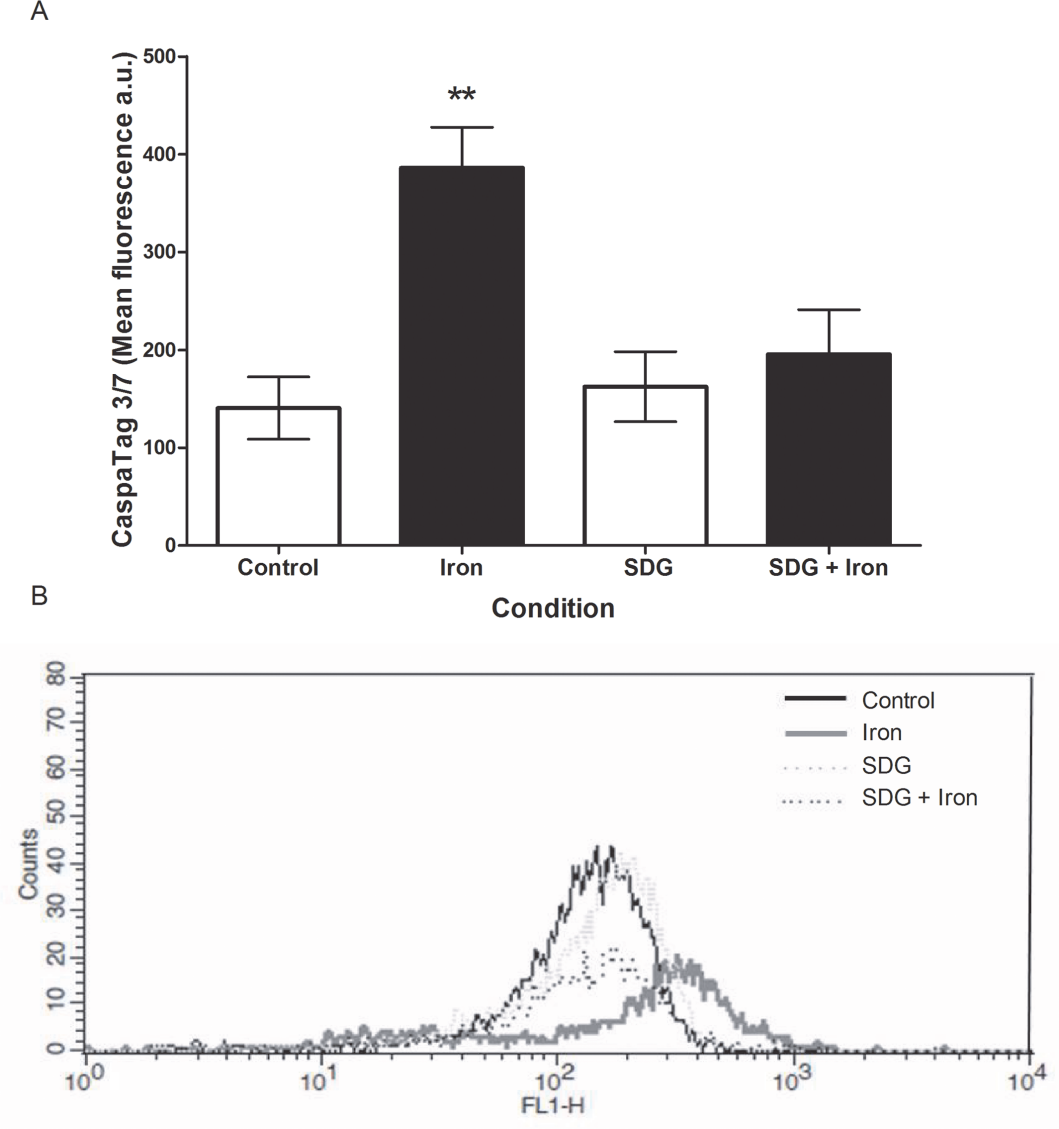
SDG decreases iron-induced caspase 3/7 activity in H9c2 cells. Active caspases 3/7 in control, 50 μM iron-treated, 500 μM secoisolariciresinol diglucoside (SDG), and SDG pre-treatment + iron-treated H9c2 cells. Caspase levels were determined via the CaspaTag 3/7 assay and measured via flow cytometry. Data is expressed as mean fluorescence arbitrary units (a.u.) (A). The representative histogram of cell count versus FL1 fluorescence (B). (** = p < 0.01 versus control, *n* = 3).

To investigate the potential cytotoxicity of the observed iron-induced ROS production, the activity of apoptotic proteins (caspases 3 and 7) was assessed using the CaspaTag assay and measured via flow cytometry. Iron caused a significant increase (~174%, p < 0.01) in apoptosis (Fig 6*A*) as indicated by increased mean FL1 fluorescence (representative histogram; Fig 6*B*) versus control. Given the observed SDG-mediated prevention of iron-induced ROS elaboration, the potential cytoprotective effect of SDG was investigated. Pre-treatment with SDG prevented the significant increase in iron-induced apoptosis, maintaining it at control levels (Fig 6*A* and 6*B*). This finding suggests that the observed iron-induced cytotoxicity was largely mediated by ROS and/or inflammation, and that SDG was effective in preventing both the iron-induced cellular damage and cell death.

### Effect of SDG on FOXO3a, Bax and Bcl2 protein levels

**Fig 7:**
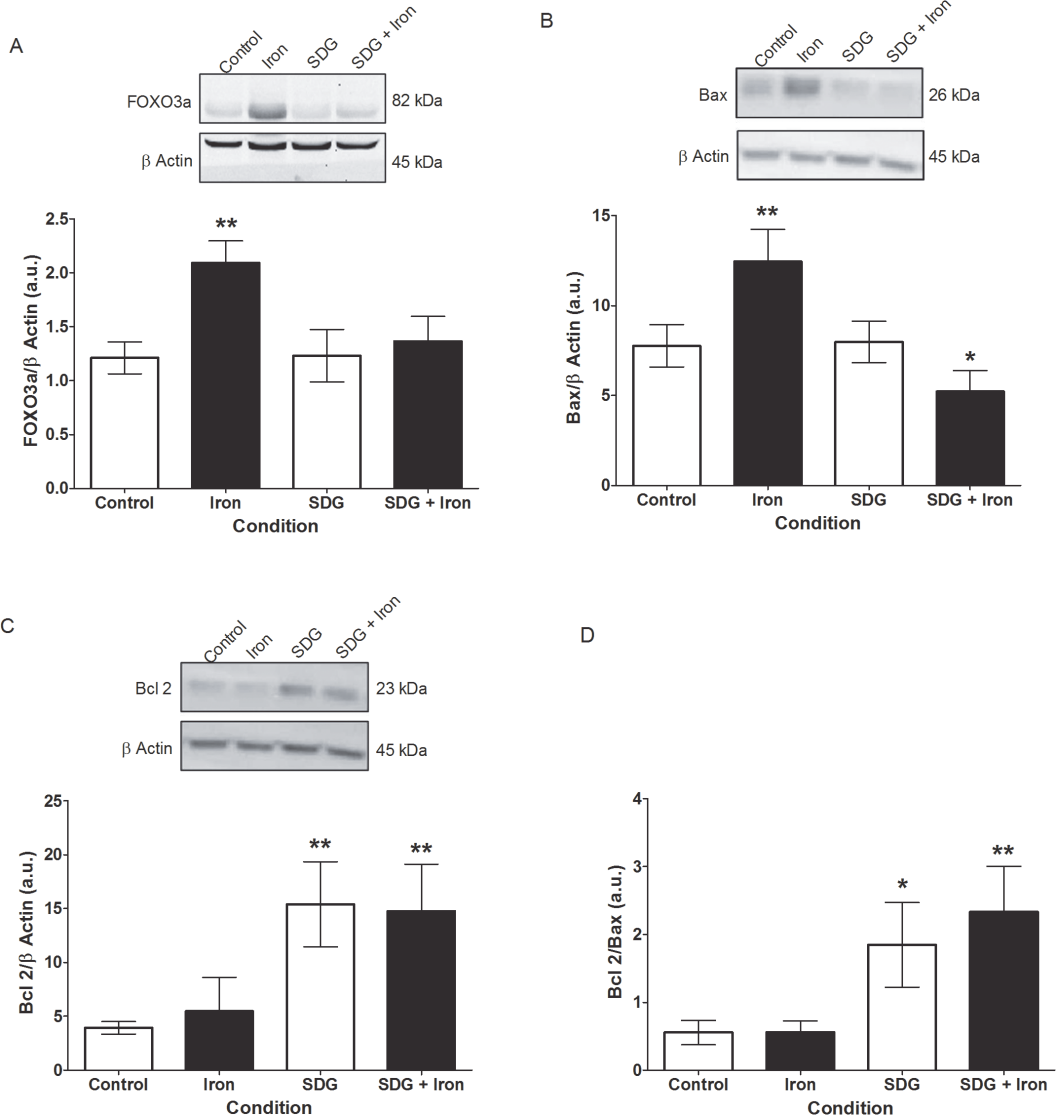
SDG decreases iron-induced apoptosis in H9c2 cells. Forkhead box O (FOXO)3a (A), Bax (B) Bcl2 (C) protein levels and Bcl2/Bax ratio (D) in control, 50 μM iron-treated, 500 μM secoisolariciresinol diglucoside (SDG), and SDG pre-treatment + iron-treated H9c2 cells. Protein levels were measured via immunoblotting. Data is expressed as protein/β-actin arbitrary units (a.u) (** = p < 0.01 versus control; * = p < 0.05 versus control, *n* = 4).

To further investigate the iron-induced apoptosis FOXO3a, Bax, and Bcl2 protein expression were assessed via immunoblotting. FOXO3a and Bax protein expression were significantly increased after 24-hour iron treatment (~0.9 fold, p < 0.01 and ~4.7 fold, p < 0.01 respectively) when compared to control (Fig 7*A* and 7*B*). Pre-treatment with SDG prevented the increase in iron-induced FOXO3a and Bax protein expression, maintaining it at control levels (Fig 7*A* and 7*B*). Bcl2 protein expression did not change significantly after 24-hour iron treatment when compared to control (Fig 7*C*). Pre-treatment with SDG led to a significant increase in Bcl2 protein expression when compared to control (~10.8 fold, p < 0.01) (Fig 7*C*). Bcl2/Bax ratio did not change in iron treated cells compared to control but was significantly increased when cells were treated with SDG alone and pre-treated with SDG prior to iron treatment when compared to control (~1.3 fold, p < 0.05; ~1.8 fold, p < 0.01 respectively) (Fig 7*D*). These findings further suggest SDG was effective in attenuating iron-induced cell death.

### Effect of SDG on p70S6K1 and AMPK protein levels

**Fig 8:**
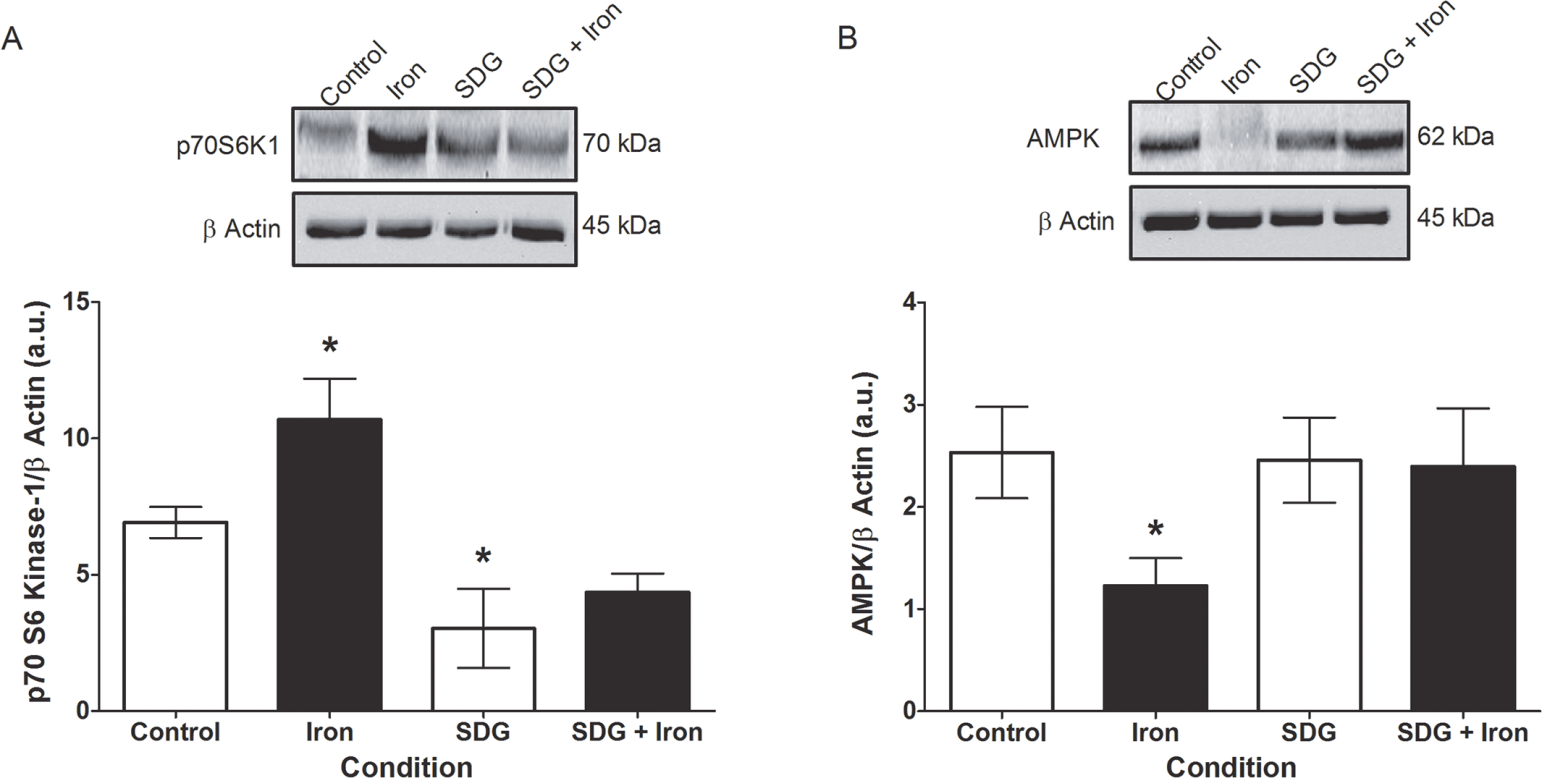
SDG decreases p70S6K1 and increases AMPK protein expression in iron treated H9c2 cells. p70S6 Kinase 1 (p70S6K1) (A) and AMP-activated protein kinase (AMPK) (B) protein levels in control, 50 μM iron-treated, 500 μM secoisolariciresinol diglucoside (SDG), and SDG pre-treatment + iron-treated H9c2 cells. Protein levels were measured via immunoblotting. Data is expressed as protein/β-actin arbitrary units (a.u) (* = p < 0.05 versus control, *n* = 3).

p70S6K1 protein expression was assessed via immunoblotting and was significantly increased (~3.8 fold, p < 0.05) after 24-hour iron treatment when compared to control (Fig 8*A*). Treatment with SDG alone reduced p70S6K1 protein expression significantly compared to control (~3.9 fold p < 0.05). Pre-treatment with SDG prevented the increase in iron-induced p70S6K1 protein expression (Fig 8*A*). AMPK protein expression was significantly decreased after iron treatment (~1.3 fold, p < 0.05) when compared to control levels (Fig 8*B*). Pre-treatment with SDG abolished the decrease in iron-induced AMPK protein expression, maintaining it at control levels (Fig 8*B*).

## Discussion

In the present study, we found that pre-treatment with SDG prevented a significant increase in iron-induced apoptosis, suggesting that the observed iron-induced cytotoxicity is largely mediated by oxidative stress and inflammation. Importantly, this also indicates that SDG is effective in mitigating iron-induced cellular damage and death, representing the first report of a cardioprotective role for SDG in a cardiac iron overload condition.

Iron overload is one of the most common causes of myocardial injury and diastolic heart failure that is directly attributable to oxidative stress [2, 5–8]. Studies conducted *in vivo* have demonstrated an increase in ROS even after short-term iron exposure [24, 25]. In agreement with this concept, our results demonstrated a pronounced increase in oxidative stress upon iron treatment of cardiac H9c2 cells.

SDG is an antioxidant present in flaxseed and is known to decrease the production of inflammatory mediators and scavenge the ROS, specifically the hydroxyl radical [20]. Studies have shown that SDG prevents the development of hypercholesterolemic atherosclerosis [21], induces angiogenesis-mediated cardioprotection [22], and prevents the development of type 1 and type 2 diabetes [19, 23]. Although several studies have investigated SDG, none have explored its antioxidant potential in a cardiac iron overload.

Increased ROS have been implicated in initiating harmful events including DNA damage, lipid peroxidation and activation of MMPs which contribute to cardiovascular remodeling and dysfunction [9, 11, 26, 27]. Previous studies using the same cardiac cell line found that iron overload causes progressive loss of intact mitochondrial DNA, decreased expression of respiratory chain subunits encoded by mitochondrial and diminished respiratory function. They also reported that iron-mediated cytotoxicity involves ROS generated by the mitochondrion itself because cells lacking mitochondrial DNA were remarkably tolerant of iron overload [24]. In our study ROS production was shown to increase in 50 μM iron treated cells as shown with the CM-H_2_DCFDA assay. Pre-treatment with SDG prevented the significant increase in iron-induced ROS generation, reducing it to control levels. Moreover, iron induced decrease in SOD concentration was abrogated by pre-treatment with SDG, thereby suggesting an antioxidant potential of SDG in iron overload condition.

Cardiovascular diseases are associated with inflammation and cytokine modulation [7, 9, 11, 28], and chronic heart failure is often characterized by elevated pro-inflammatory cytokine expression [9, 11, 28, 29]. TNF-α and IL-10 were found to be elevated in iron overloaded patients with thalassemia major [30]. Among its many effects, TNF-α is an initiator of the extrinsic apoptosis pathway [31]. IL-10 is an anti-inflammatory cytokine known to down-regulate the production of TNF-α, and it has similarly been detected in failing myocardium [32, 33]. In the iron overload condition there was a significantly higher expression of pro and anti-inflammatory cytokines. Taken together, these data suggest a strong inflammatory response in iron overload. Pre-treatment with SDG counteracted the significant increase in iron-induced TNF-α and IL-10 expression, maintaining it at control levels, while IFNγ expression significantly decreased when compared to iron treated cells. These data suggest SDG is capable of preventing the increase in inflammation in iron treated cells. Dietary flax seed is reported to suppress the production of TNF-α in hypercholesterolemic atherosclerosis in rabbits [21].

Matrix metalloproteinases participate in tissue remodelling in cardiovascular diseases associated with enhanced oxidative stress [34]. MMP-2 and MMP-9 are known to play key roles in various cardiac disease conditions [35–37]. MMP activity has been shown to be regulated at multiple levels including its upregulation by TNF- α [13]. A recent study has demonstrated that TNF-α can trigger the expression and activation of MMPs via superoxide production [13]. Increased oxidative stress and inflammation observed in our study may act as a trigger for increased expression of MMP 2 and 9. A previous study demonstrated that H9c2 cardiomyocytes exposed to H_2_O_2_ induced oxidative stress, exhibited increased MMP-2 activity, leading to cleavage and activation of the apoptotic protein, glycogen synthase kinase-3β [34]. Incubation with doxorubicin, an antitumor agent that causes heart damage, leads to an increase in MMP-2 and 9 expression and activation in H9c2 cells [36]. In the present iron overload condition, we demonstrated increased mRNA expression of both MMP-2 and 9, which was associated with increased oxidative stress and inflammation. This observation is consistent with the significant cardiac damage caused by iron overload, whereby matrix remodelling plays a role in the pathophysiology of cardiac dysfunction. Pre-treatment with SDG counteracted the significant increase in iron-induced MMP-2 and 9 expressions, maintaining it at control levels, indicating that SDG can have an effect on matrix components.

Two independent pathways may lead to cardiomyocyte apoptosis; intrinsic and extrinsic apoptosis. Both pathways end with the cleavage and activation of executioner caspases 3 and 7 [38]. We showed a significant increase in active caspase 3/7 in iron treated cells. Pre-treatment with SDG attenuated the significant increase in iron-induced apoptosis, maintaining it at near control levels. To further investigate the iron-induced apoptosis FOXO3a, Bax, and Bcl2 protein expression were assessed via immunoblotting. FOXO3a acts as a transcription factor known to play important roles in the regulation of apoptosis, while Bax can lead to the release of cytochrome c [39, 40]. FOXO3a and caspase 3 were activated in rat cardiac microvascular endothelial cells subsequent to myocardial ischemia/reperfusion injury. Moreover, FOXO3a inhibition led to a decrease in apoptosis via decrease in caspase 3 activation [41]. In our study FOXO3a and Bax protein expression were significantly increased after 24-hour iron treatment when compared to control. Pre-treatment with SDG prevented the significant increase in iron-induced FOXO3a and Bax protein expression. Bcl2 protein expression did not change significantly after the 24-hour iron treatment when compared to control. Pre-treatment with SDG led to a significant increase in Bcl2 protein expression when compared to control and iron treatment. Bcl2/Bax ratio increased after treatment with SDG alone and pre-treatment with SDG prior to iron treatment when compared to both control and iron treated cells. These data indicate concomitant iron-induced increases in apoptosis and a better understanding into the anti-apoptotic role of SDG in cardiac iron overload condition.

Cardiac remodeling occurs in response to oxidative stress, which involves activation of p70S6K1 [14]. A study showed that treatment of cardiomyocytes with H_2_O_2_ led to an increase of p70S6K1 activity as early as 30 minutes after treatment [14]. Another study showed that resveratrol reduces hypertrophic growth of the myocardium by inhibiting p70S6K and enhancing the LKB1/AMPK pathway [42]. AMPK reserves cellular energy content and serves as a key regulator of cell survival in response to pathological stress. AMPK activation in a hypertrophic mouse model has been shown to be cardioprotective by blocking p70S6K1 and attenuating mTOR and ERK1/2 activation [16]. In our study p70S6K1 protein expression was assessed via immunoblotting and was significantly increased after 24-hour iron treatment when compared to control. Pre-treatment with SDG negated the significant increase in iron-induced p70S6K1 protein expression, maintaining it at control levels. This decrease in iron-induced p70S6K1 protein expression suggests that SDG may play a role in cardiac remodeling by modulating levels of p70S6K1. AMPK protein expression was found to be significantly decreased after iron treatment when compared to control levels. Pre-treatment with SDG prevented the significant decrease in iron-induced AMPK protein expression. This suggests a cardioprotective role for SDG in cardiac iron overload.

In conclusion, iron overload in cardiac H9c2 cells caused increased ROS, apoptosis, and inflammation. SDG abrogated the observed increases in ROS and apoptosis, suggesting a previously unknown cardioprotective role for this flaxseed in cardiac iron overload condition.
